# Performance of the Disc Diffusion Method, MTS Gradient Tests and Two Commercially Available Microdilution Tests for the Determination of Cefiderocol Susceptibility in *Acinetobacter* spp.

**DOI:** 10.3390/microorganisms11081971

**Published:** 2023-07-31

**Authors:** Katy Jeannot, Susie Gaillot, Pauline Triponney, Sylvain Portets, Valentin Pourchet, Damien Fournier, Anaïs Potron

**Affiliations:** 1 Laboratoire de Bactériologie, CHU de Besançon, F-25000 Besançon, France; 2 Centre National de Référence de la Résistance aux Antibiotiques, Laboratoire Associé, Centre Hospitalier Universitaire de Besançon, F-25000 Besançon, France; 3 CNRS, UMR 6249, Chrono-Environnement, Université de Franche-Comté, F-25000 Besançon, France

**Keywords:** cefiderocol, *Acinetobacter* spp., susceptibility testing

## Abstract

Cefiderocol is a siderophore-conjugated cephalosporin with potent activity against multidrug-resistant Gram-negative pathogens including *Acinetobacter baumannii*. The aim of this study was to evaluate cefiderocol testing methods on a relevant collection of 97 *Acinetobacter* spp. isolates. Commercialized broth microdilution methods (ComASP^®^, Liofilchem and UMIC^®^, Bruker), MIC test strips (Liofilchem) and disc diffusion using discs of three different brands (Mast Diagnostic, Liofilchem and Oxoid—Thermo Fisher Scientific) were compared with the broth microdilution reference method. None of the methods tested fulfilled acceptable criteria (essential agreement [EA] ≥ 90%; bias = ±30%) but both BMD methods achieved acceptable categorical agreement rates (CA = 95.9% [93/97, 95% CI 89.9–98.4] and CA = 93.8% [91/97, 95% CI 87.2–97.1] for ComASP^®^ and UMIC^®^, respectively) and bias < 30% (−7.2% and −25.2% for ComASP^®^ and UMIC^®^, respectively). The use of MIC gradient testing is strongly discouraged due to misclassification of 55% (*n *= 23/42) of resistant strains. Finally, the disc diffusion method could be used to rapidly screen for susceptible strains by setting a critical diameter of 22 mm.

## 1. Introduction

Carbapenem-resistant *Acinetobacter baumannii-calcoaceticus* (CRAB) complex is one of the leading causes of healthcare-associated infections worldwide [1,2]. Given the limited therapeutic options available, CRAB have been included by the World Health Organisation (WHO) in the global priority list of antibiotic-resistant bacteria, for which antibiotics are urgently needed [3]. Recently, cefiderocol, a siderophore-conjugate cephalosporin, was shown to have an excellent in vitro activity against Gram-negative bacilli, including carbapenem-resistant *Acinetobacter baumannii* isolates [4,5,6]. However, due to limited clinical data, IDSA and ESCMID experts recommend caution in the use of cefiderocol for the treatment of moderate to severe CRAB infections. This molecule should indeed be administered as part of a combination regimen and after other therapeutic alternatives have been exhausted [7,8]. While the reference method for determining cefiderocol susceptibility is the broth microdilution (BMD) method using an iron-depleted cation-adjusted Mueller–Hinton broth (ID CA-MHB) [9], this method is not convenient for routine clinical microbiology laboratories. In addition, the reading of BMD requires expertise as the value of MIC corresponds to the first well in which the reduction in growth corresponds to a button of <1 mm or is replaced by the presence of light haze/faint turbidity [9]. Other susceptibility testing methods have been successively commercialized, including MIC test strips (MTS) and discs, which can be used with regular Mueller–Hinton agar (MHA), and UMIC^®^ and ComASP^®^ microplates using ID CA-MHB. The performance of some of those methods has been evaluated for *Enterobacterales* and *Pseudomonas aeruginosa*, showing that microplates should be an alternative to the reference BMD method and that MIC strips may not be used [10,11]. In addition, several sources reported large variations in inhibitory zone diameters, depending on the disc and MHA brands, for QC and clinical strains [10,12]. Cefiderocol susceptibility testing appears particularly tricky in *A. baumannii* regarding the reproducibility and accuracy of both cefiderocol BMD and disc diffusion methods [13,14]. However, providing accurate information to clinicians about cefiderocol susceptibility of multidrug-resistant *A. baumannii* isolates is crucial in view of the few therapeutic alternatives available. Here, we evaluated the performances of two microdilution methods, disc diffusion and MIC test strips, compared to the BMD reference method on a relevant collection of *Acinetobacter* spp. clinical isolates.

## 2. Materials and Methods

### 2.1. Bacterial Isolates

Ninety-seven *Acinetobacter* spp. strains isolated from several clinical samples between 2012 and 2022 were selected from 59 French medical laboratories (Table S1). Those strains were referred to the French National Reference Centre for Antibiotic Resistance (NRC-AR) for analysis of their resistance mechanisms to antibiotics. The strains selected for this study were genetically diverse, belonging to 27 different STs according to the Pasteur Schema, with ST2 and ST1 being the most represented STs (37% and 12% of *A. baumannii* strains, respectively). All strains showed decreased susceptibility to at least one carbapenem (meropenem (MIC > 2 mg/L) and/or imipenem (MIC > 2 mg/L)) according to the current EUCAST recommendations [15]. Among the 97 selected strains, 70 produced only one carbapenemase, being an OXA-type carbapenem-hydrolysing beta-lactamase in 58 strains, an NDM-type enzyme in 11 strains and an IMP-type enzyme in one strain. Twenty-two isolates possessed two carbapenemase-encoding genes (frequently NDM-1 and OXA-23, *n* = 16). An ESBL was identified in 17 strains, of which 16 co-produced a carbapenemase (Table S1). Finally, one strain produced only a GES-11 ESBL, and 5 overproduced their intrinsic cephalosporinase (Table S1). Quality controls using wild-type *P. aeruginosa* strain ATCC 27853 were included in each series of experiments (*n* = 6).

### 2.2. Cefiderocol Susceptibility Testing

#### 2.2.1. Broth Microdilution Reference Method (BMD)

Reference MIC values were determined using an iron-depleted and cation-adjusted Mueller–Hinton broth (ID-CAMHB) and a titrated powder of cefiderocol (MedChemExpress, Sollentuna, Sweden) as described previously and according to the recommendation of the Clinical and Laboratory Standards Institute [10,16]. Briefly, a commercial Mueller–Hinton broth (Becton Dickinson, Le Pont-de-Claix, France) was treated with a Chelex resin (Bio-Rad Laboratories, Hercules, CA, USA) and then supplemented with divalent cations (Mg^2+^, Ca^2+^, Zn^2+^). The concentration of iron and divalent cations in the broth was measured by flame spectrometry (QUALIO, Besançon, France). All ID-CAMHB batches prepared contained less than 0.03 mg/L iron. Cefiderocol MIC values in microplates were read by 3 operators and interpretated according to EUCAST PK-PD breakpoints (susceptible, ≤2 mg/L and resistant > 2 mg/L). At least two MIC determinations using the reference method was performed for each strain, and a third determination was carried out in case of variation of more than one dilution or difficulty of reading.

#### 2.2.2. Other Susceptibility Testing Methods

The UMIC^®^ cefiderocol unit test (Bruker, Wissembourg, France) and the microplate ComASP^®^ (two tests by microplate) (Liofilchem, Roseto degli Abruzzi, Italy, distributed by I2A, Montpellier, France) were used according to the manufacturer’s recommendations. Briefly, both tests are based on the inoculation of precoated wells containing cefiderocol at concentrations ranging from 0.03 to 32 mg/L for the UMIC^®^ test and 0.008 to 128 mg/L for the ComASP^®^ method. The UMIC^®^ strips were inoculated with 100 μL of a suspension prepared by adding 25 μL of a 0.5 McFarland adjusted bacterial suspension to 5 mL of iron-depleted CAMHB supplied by the manufacturer. The ComASP^®^ method requires an additional step, which is a 1:20 dilution of the 0.5 McFarland bacterial suspension in a saline solution. As recommended by the EUCAST, the MIC was read as the first well in which the reduction of growth corresponds to a button of <1 mm or is replaced by the presence of light haze/faint turbidity [9]. Cefiderocol MTS from Liofilchem (Roseto degli Abruzzi, Italy, distributed by I2A, Montpellier, France) were tested with Becton Dickinson Mueller–Hinton agar (Heidelberg, Germany) as recommended by the supplier. All MIC values were read separately by 3 operators and the values obtained with strips were rounded to the upper highest 2-fold dilution, following the manufacturer’s instructions. Disc diffusion assays were carried out following the EUCAST guidelines (https://eucast.org/ (accessed on 21 June 2023)) with cefiderocol discs loaded at 30 micrograms from 3 manufacturers (Mast Diagnostic, Merseyside, UK; Liofilchem, Roseto degli Abruzzi, Italy; and Oxoid—Thermo Fisher Scientific, Basingstoke, UK) and Becton Dickinson MHA. This latter was chosen because the inhibition zone diameters for the *P. aeruginosa* ATCC 27853 quality control strain in previous study were within the acceptable range for this MHA manufacturer [10,17]. Colonies within inhibition zones were taken into account and diameters were measured independently by 3 operators and interpreted according to the recommendations of the EUCAST (strain categorized as susceptible if inhibition zone diameter ≥ 17 mm and resistant if inhibition zone diameter < 17 mm).

### 2.3. Data Analysis

Categorical agreement (CA), essential agreement (EA), inferior and superior bias were calculated according to ISO 20776-2:2021 standards, with the BMD method and EUCAST PK-PD breakpoints as reference. Percentages ≥ 90% for EA, and a difference for bias +/− 30% were considered as acceptable.

## 3. Results

### 3.1. Broth Microdilution

The MICs values of cefiderocol ranged from 0.06 mg/L to >16 mg/L with a MIC_50_ = 2 mg/L and a MIC_90_ > 16 mg/L with the BMD reference method. Forty-two strains (43.3%) were classified as resistant to cefiderocol, according to the EUCAST PK-PD breakpoints (Table S1 and Figure S1). Interestingly, the MIC values of cefiderocol for the quality control strain *P. aeruginosa* ATCC 27853 were within the acceptable range for all microdilution tests, except for the UMIC^®^ test, where one value was out of the acceptable range (Figure S2).

**Figure 1:**
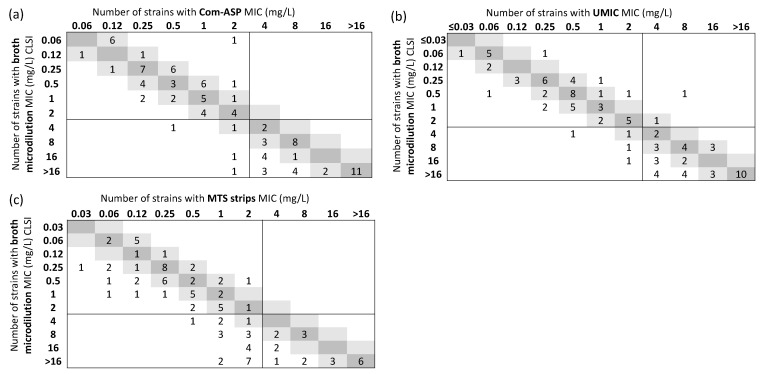
Correlation between cefiderocol MIC determined by broth microdilution reference method, (**a**) and by ComASP^®^, (**b**) by UMIC^®^ and (**c**) by MTS gradient test strips. The number of strains with MIC corresponding to the broth microdilution method, and 1-log_2_ dilution are highlighted in dark and light grey areas, respectively. EUCAST 2023 PK-PD breakpoint is indicated by solid black lines.

Compared with BMD reference method, the ComASP^®^ and UMIC^®^ microdilution tests displayed a CA rate higher than 90% (CA = 95.9%, *n *= 93/97, 95% CI 89.9–98.4 for ComASP^®^ and CA = 93.8%, 95% CI 87.2–97.1 for UMIC^®^) (Figure 1a,b and Table 1). However, both methods did not achieve an acceptable 90% EA rate (81.4%, *n *= 79/97, 95% CI 72.6–87.9 for ComASP^®^ and 78.4%, 95% CI 69.2–85.4 for UMIC^®^). While the UMIC^®^ test tended to provide lower MIC values, as illustrated by the high percentage of inferior bias (−42.3%), the lower (−36.1%) and higher (+28.9%) bias values were significant for the ComASP^®^ test (Figure 1a,b and Table 1). A total of four strains were classified as susceptible by each method, whereas they were classified as resistant by the reference method. Of these, two were misclassified by both BMD methods (17A1955 and 22A3377, which produced the NDM-1 and OXA-23 enzyme, respectively). 

### 3.2. Gradient Strips

While the QC were within acceptable limits, none of the calculated rates (CA = 76.3%, 95% CI 66.9–83.7; EA = 59.8%, 95% CI 49.8–69 and bias −48.4%) were acceptable for gradient strips (Figure 1c and Figure S2, Table 1). The high value of inferior bias (−62.9%) indicated that the method underestimated the MIC values in comparison with the BMD reference method. Thus, 55% of cefiderocol-resistant strains (*n *= 23/42) were misclassified as susceptible by the MTS strips. 

### 3.3. Disc Diffusion

**Figure 2:**
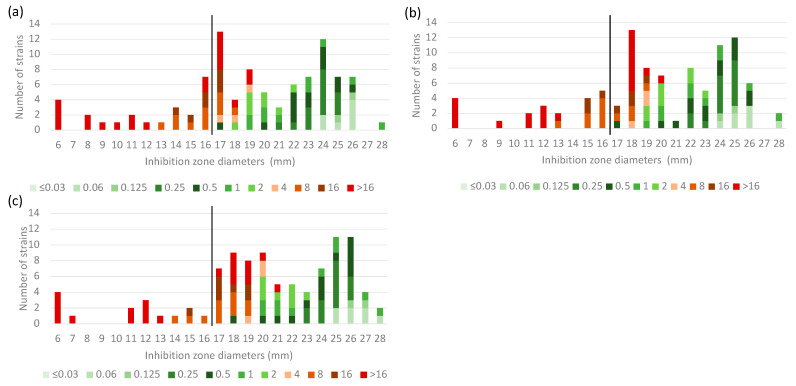
Distribution of inhibition zone diameter according to the MIC (mg/L) determined by broth microdilution reference method. Each isolate was tested with cefiderocol discs (30 μg) on Becton Dickinson Mueller-Hinton agar from manufacturers (**a**) Mast, (**b**) Liofilchem and (**c**) Oxoid. EUCAST 2023 PK-PD breakpoint is indicated by solid black line (17 mm).

While the inhibition zone diameters for the *P. aeruginosa* ATCC 27853 QC strain were all within the acceptable range (23–29 mm) regardless of disc supplier (Mast, Liofilchem and Oxoid) (Figure S3), they did not meet the acceptability criteria (±1 mm of the target value) defined by the EUCAST in its warning published in August 2022 [12]. The disc diffusion method did not achieve an acceptable CA rate, which varied from 72.2% to 81.4% (Figure 2, Table 1). Of note, ten isolates (eight resistant and two susceptible to cefiderocol) showed microcolonies in the zone of inhibition for the three types of disc considered for reading. Whereas the disc diffusion method accurately categorized the cefiderocol susceptible strains, the proportion of misclassified strains among resistant isolates (*n *= 42) was 42.9% (*n *= 18/42), 50% (*n *= 21/42) and 64.3% (*n *= 27/42), for MAST, Liofilchem and Oxoid discs, respectively. These errors were not associated with a ß-lactam resistance mechanism, disc reading difficulties, or a subpopulation of strains belonging to an identical genetic background.

## 4. Discussion

Using a strain collection set up to be representative of the current epidemiology of multidrug-resistant *Acinetobacter* spp. strains, none of the methods tested fully met the criteria set by the International Standards Organization (ISO 20776-2:2021; EA ≥ 90%, difference of bias ± 30%). Both BMD methods evaluated (UMIC^®^ and ComASP^®^ tests) showed high CA rate (≥90%) and acceptable differences of bias but EA rates remained below the 90% threshold. The UMIC^®^ test tended to underestimate MIC values (bias inferior −42.9%) in *Acinetobacter* spp. Recently, the UMIC^®^ method, tested on 44 *A. baumannii* isolates (of which 13 were resistant) showed similar performance (CA of 90.9%, EA of 84.1%) but lower inferior bias (−11.4%) [18]. Among the 42 cefiderocol-resistant strains of our study, three strains, producing an NDM-like enzyme alone (*n *= 2) or in combination with an OXA-23 enzyme (*n *= 1), and one OXA-72-producing isolate were misclassified as susceptible by one of the BMD methods (ComASP^®^ or UMIC^®^), all with a MIC at 2 mg/L. In addition, both methods classified two strains as susceptible, one OXA-23 and one NDM-1, the latter with an MIC of 2 mg/L by both methods. However, it is important to note that independent of quality control results, a poor reproducibility of BMD and disc diffusion methods has been reported for *Acinetobacter* spp. strains [14]. In fact, some strains initially classified as resistant by the BMD method were found to be fully susceptible on repeat testing [14]. In our study, the determination of the MIC of cefiderocol by BMD was repeated whenever a discrepancy was found between the BMD method and the test evaluated at more than one dilution, or resulted in a change in the categorization of the strain. In total, 16 strains (16.5%), initially classified as resistant (*n* = 3) or susceptible (*n* = 13), changed category on repeat testing. In addition, as previously mentioned, the interpretation of the BMD tests is often complicated by the presence of a haze or trail [14]. Indeed, more than a third (31%) of the strains tested in our collection were difficult to interpret, and the MIC of cefiderocol was determined after several tests.

The results obtained in this study using cefiderocol MTS gradient strips on clinical isolates of *Acinetobacter* spp. did not differ from those previously observed for *Enterobacterales* and *P. aeruginosa* isolates, as they dramatically underestimated the cefiderocol MIC values [10,11]. At present, their use is strongly discouraged because of the risk of failing to identify a cefiderocol-resistant strain. Considering the acceptability criteria for *P. aeruginosa* ATCC 27853 quality control strain values published by EUCAST in August 2022 (±1 mm from the target value), one to five values out of six experiments were above the acceptable limit, indicating that it would be difficult to interpret cefiderocol disc diffusion using these criteria [12]. From our study, it appears that the cut-off diameter corresponding to the PK/PD breakpoint established by EUCAST (≥17 mm, susceptible) is not sufficiently reliable for determining cefiderocol susceptibility on *Acinetobacter* spp. clinical strains. Indeed, a large number of strains (from 18 to 27 isolates, depending on the disc tested) were categorized as susceptible, whereas they were resistant by the reference method (Figure 2). A revision of the cut-off diameter seems necessary if its use is to be maintained. However, the disc diffusion method might be used as a screening tool for cefiderocol-susceptible strains. Over 21 mm (≥22 mm), all isolates were categorized as susceptible to cefiderocol, regardless of the disc supplier (Figure 2). For strains with an inhibition zone diameter < 22 mm, we suggest to confirm the disc zone diameter by a BMD method. However, this study was conducted with only one MHA supplier (Becton Dickinson) and further investigations are required to validate this breakpoint diameter with discs from other manufacturers.

Previous studies have reported that the production of PER- and NDM-β-lactamases is associated with an increase in cefiderocol MICs [19,20]. While it is difficult to predict cefiderocol susceptibility in *Enterobacterales* and *P. aeruginosa* clinical isolates, identification of the β-lactamase resistance mechanisms in *Acinetobacter* spp. isolates can help predict cefiderocol susceptibility. In our collection, all PER-type β-lactamase-producing strains (*n* = 10) were highly resistant to cefiderocol (MIC > 16 mg/L), and more than 80% of strains producing NDM-like enzymes (*n *= 26/31) were also resistant to cefiderocol (MIC ≥ 4 mg/L). Four NDM-1-producing strains were susceptible with MIC close to the PK-PD breakpoint (MIC = 2 mg/L) (Table S1). Oxacillinase-producing strains that do not co-produce NDM-like or PER-like enzymes (*n *= 52/97) were more likely to be susceptible to cefiderocol, as 11.5% of these strains were resistant.

## 5. Conclusions

While the use of MTS strips should be strongly discouraged due to their underestimation of cefiderocol MICs, the disc diffusion method should be used as a susceptibility screening tool with a cut-off diameter of 22 mm. Although none of the commercially available BMD methods evaluated (UMIC^®^ and ComASP^®^ tests) met the acceptable criteria defined by the ISO, both showed a CA rate > 90%, allowing their use for determining the cefiderocol susceptibility of *Acinetobacter* spp. isolates. Despite the technical difficulties in assessing cefiderocol MICs, MIC determination remains necessary for carbapenem-resistant *Acinetobacter* spp., for which therapeutic alternatives are limited. The combination of a cefiderocol screening test by disc diffusion or MIC determination by BMD methods, together with rapid identification of the resistance mechanism, could help laboratories to categorize susceptible and resistant strains. Microbiologists should also alert clinicians to the difficulties in accurately determining the cefiderocol susceptibility of clinical isolates of *Acinetobacter* spp. in order to make the best therapeutic choice.

## Figures and Tables

**Table 1:** Peformance of of ComASP^®^, UMIC^®^, MTS strips, and discs for the determination of susceptibility to cefiderocol of 97 *Acinetobacter* spp. clinical isolates according to EUCAST PK-PD breakpoint in comparison with broth microdilution reference method.

		Performances (%, [*n*])				
		CA	EA	Bias high	Bias low	Difference of bias
Cefiderocol MIC determination						
	ComASP	95.9 (93)	81.4 (79)	+28.9% (22/76)	−36.1% (35/97)	**−7.2**%
	UMIC	93.8 (91)	78.4 (76)	+17.1% (13/76)	−42.3% (41/97)	**−25.2**%
	MTS strips	76.3 (74)	59.8 (58)	+14.5% (11/76)	−62.9% (61/97)	−48.4%
Disc diffusion (cefiderocol, 30 μg)						
	MAST	81.4 (79)	-	-	-	-
	Liofilchem	78.4 (76)	-	-	-	-
	Oxoid	72.2 (70)	-	-	-	-

CA, categorical agreement; EA, essential agreement; -, not applicable. The EUCAST 2023 PK/PD breakpoints were used to interpretate the results. In accordance with ISO 20776-2:2021, the acceptable rates are indicated in bold (EA ≥ 90%, and difference of bias low and high ± 30%).

## Data Availability

The data presented in this study are available upon request from the corresponding author.

## Supplementary Materials

The following supporting information can be downloaded at: https://www.mdpi.com/article/10.3390/microorganisms11081971/s1.
