# Supplementary material for: Performance of the Disc Diffusion Method, MTS Gradient Tests and Two Commercially Available Microdilution Tests for the Determination of Cefiderocol Susceptibility in Acinetobacter spp
Source: Microorganisms. 2023 Jul 31;11(8):1971. doi: 10.3390/microorganisms11081971 (PMC10458114; doi:10.3390/microorganisms11081971)
Supplement: Supplementary file 1 [file microorganisms-11-01971-s001.zip › Suppl data/Suppl figure.pptx]

## Slide 1
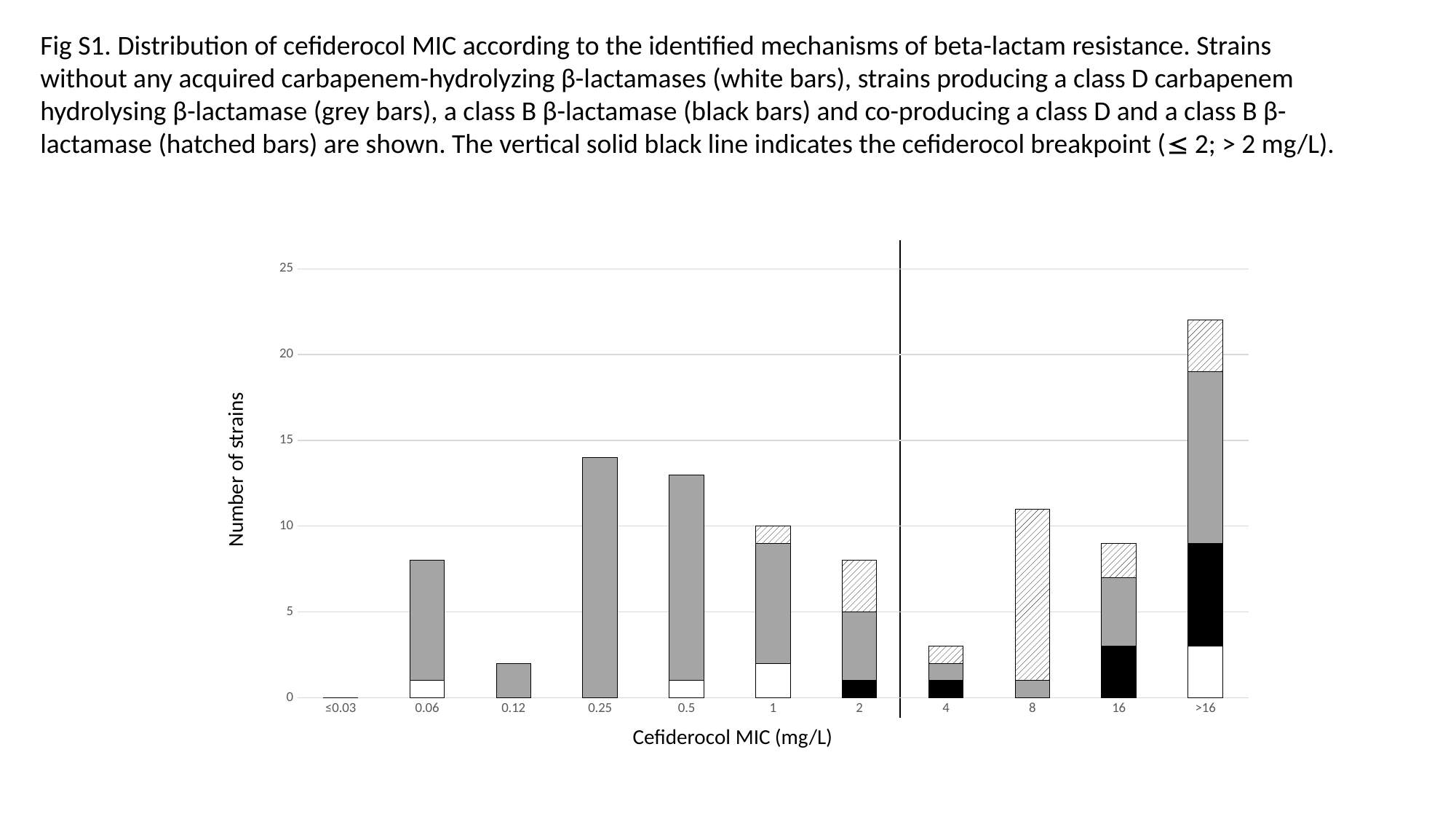

Fig S1. Distribution of cefiderocol MIC according to the identified mechanisms of beta-lactam resistance. Strains without any acquired carbapenem-hydrolyzing β-lactamases (white bars), strains producing a class D carbapenem hydrolysing β-lactamase (grey bars), a class B β-lactamase (black bars) and co-producing a class D and a class B β-lactamase (hatched bars) are shown. The vertical solid black line indicates the cefiderocol breakpoint (≤ 2; > 2 mg/L).
### Chart
| Category | Abs. Carba | Carba NDM | Carba OXA | Carba NDM + OXA |
|---|---|---|---|---|
| ≤0.03 | 0.0 | 0.0 | 0.0 | 0.0 |
| 0.06 | 1.0 | 0.0 | 7.0 | 0.0 |
| 0.12 | 0.0 | 0.0 | 2.0 | 0.0 |
| 0.25 | 0.0 | 0.0 | 14.0 | 0.0 |
| 0.5 | 1.0 | 0.0 | 12.0 | 0.0 |
| 1 | 2.0 | 0.0 | 7.0 | 1.0 |
| 2 | 0.0 | 1.0 | 4.0 | 3.0 |
| 4 | 0.0 | 1.0 | 1.0 | 1.0 |
| 8 | 0.0 | 0.0 | 1.0 | 10.0 |
| 16 | 0.0 | 3.0 | 4.0 | 2.0 |
| >16 | 3.0 | 6.0 | 10.0 | 3.0 |Number of strains
Cefiderocol MIC (mg/L)

## Slide 2
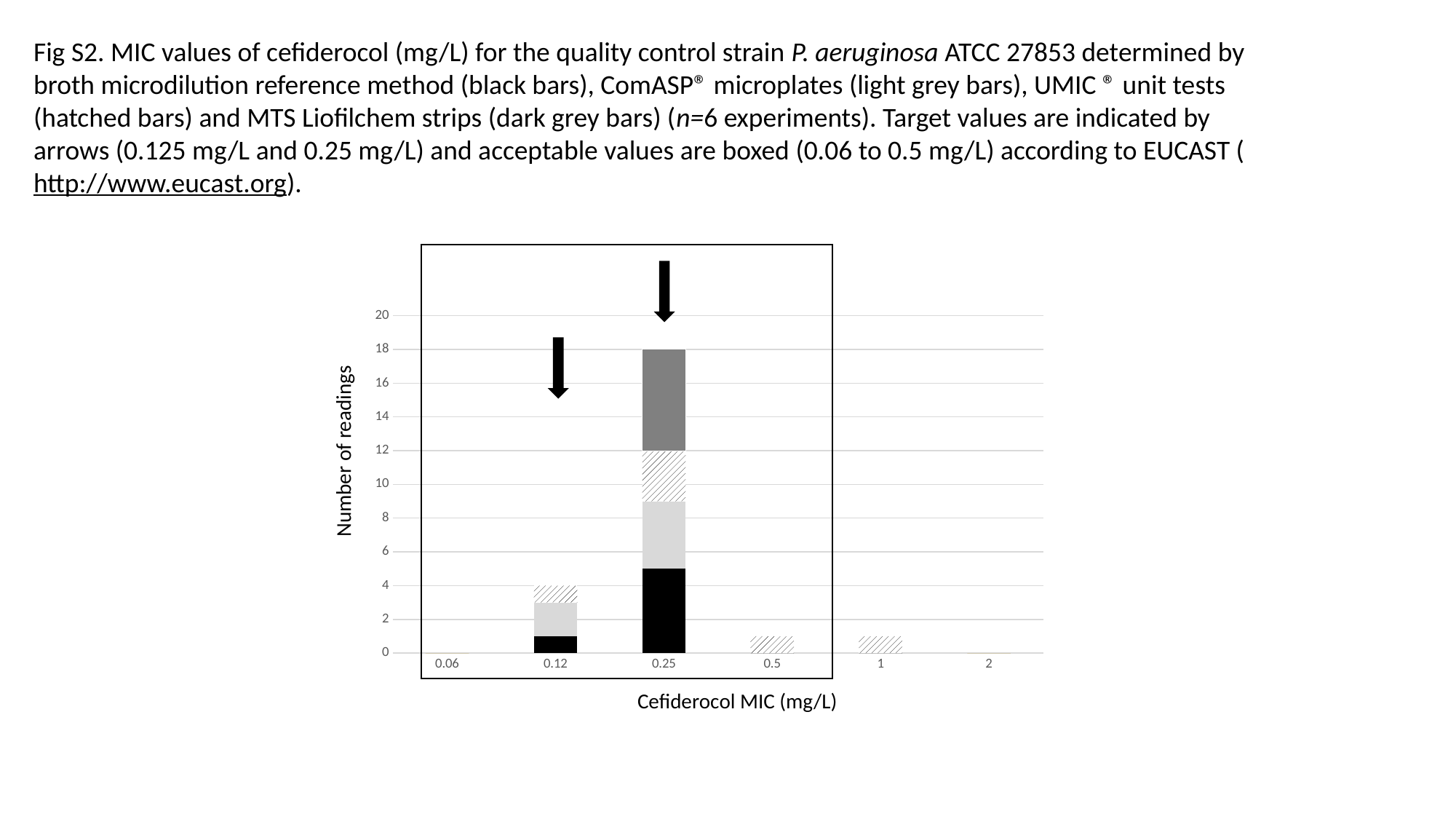

Fig S2. MIC values of cefiderocol (mg/L) for the quality control strain P. aeruginosa ATCC 27853 determined by broth microdilution reference method (black bars), ComASP® microplates (light grey bars), UMIC ® unit tests (hatched bars) and MTS Liofilchem strips (dark grey bars) (n=6 experiments). Target values are indicated by arrows (0.125 mg/L and 0.25 mg/L) and acceptable values are boxed (0.06 to 0.5 mg/L) according to EUCAST (http://www.eucast.org).
### Chart
| Category | CLSI | ComASP | UMIC | MTS |
|---|---|---|---|---|
| 0.06 | 0.0 | 0.0 | 0.0 | 0.0 |
| 0.12 | 1.0 | 2.0 | 1.0 | 0.0 |
| 0.25 | 5.0 | 4.0 | 3.0 | 6.0 |
| 0.5 | 0.0 | 0.0 | 1.0 | 0.0 |
| 1 | 0.0 | 0.0 | 1.0 | 0.0 |
| 2 | 0.0 | 0.0 | 0.0 | 0.0 |
Number of readings
Cefiderocol MIC (mg/L)

## Slide 3
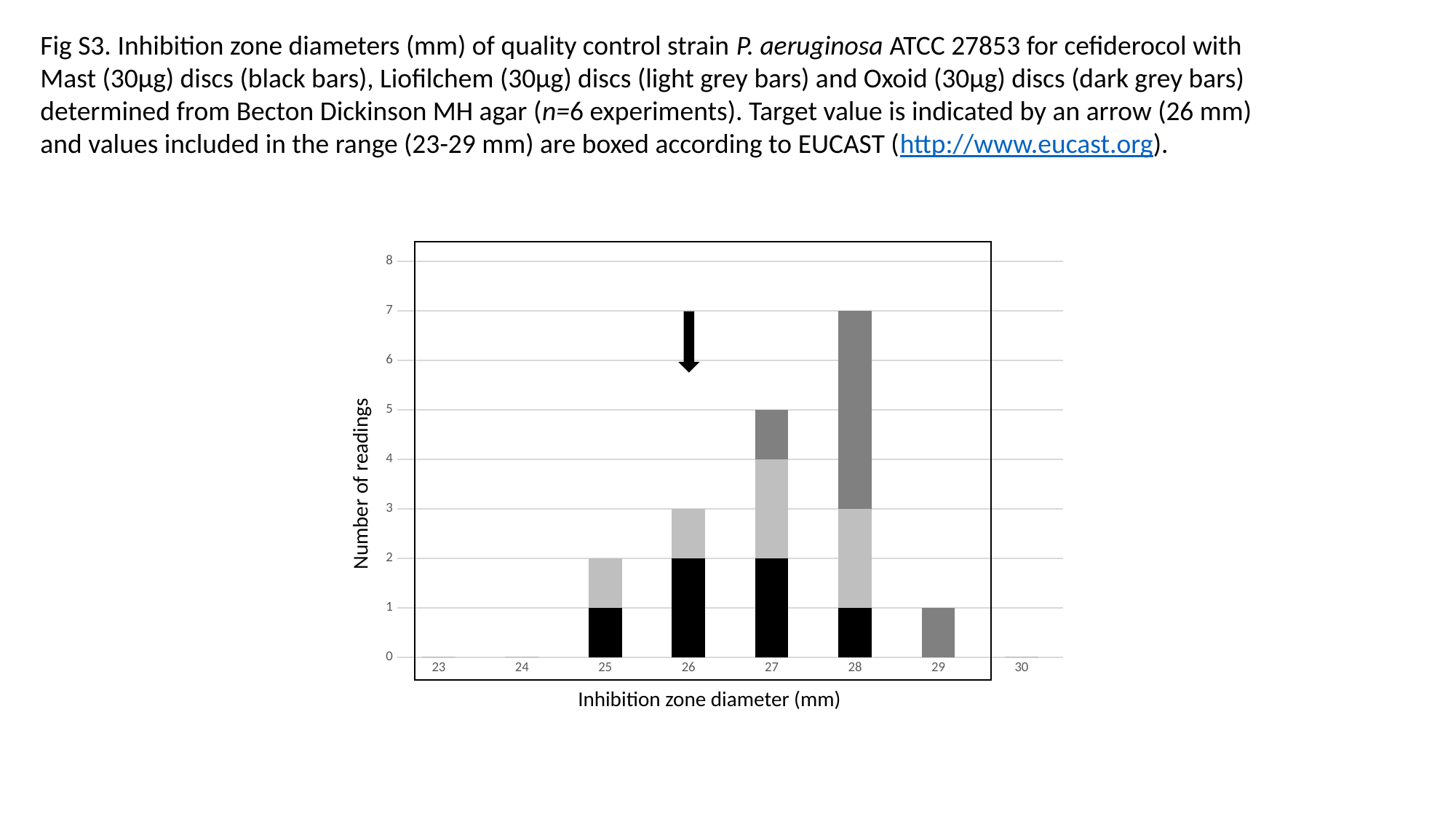

Fig S3. Inhibition zone diameters (mm) of quality control strain P. aeruginosa ATCC 27853 for cefiderocol with Mast (30µg) discs (black bars), Liofilchem (30µg) discs (light grey bars) and Oxoid (30µg) discs (dark grey bars) determined from Becton Dickinson MH agar (n=6 experiments). Target value is indicated by an arrow (26 mm) and values included in the range (23-29 mm) are boxed according to EUCAST (http://www.eucast.org).
### Chart
| Category | mast | liofilchem | oxoid |
|---|---|---|---|
| 23 | 0.0 | 0.0 | 0.0 |
| 24 | 0.0 | 0.0 | 0.0 |
| 25 | 1.0 | 1.0 | 0.0 |
| 26 | 2.0 | 1.0 | 0.0 |
| 27 | 2.0 | 2.0 | 1.0 |
| 28 | 1.0 | 2.0 | 4.0 |
| 29 | 0.0 | 0.0 | 1.0 |
| 30 | 0.0 | 0.0 | 0.0 |
Number of readings
Inhibition zone diameter (mm)
